# RKB: a Semantic Web knowledge base for RNA

**DOI:** 10.1186/2041-1480-1-S1-S2

**Published:** 2010-06-22

**Authors:** Jose Cruz-Toledo, Michel Dumontier, Marc Parisien, François Major

**Affiliations:** 1 Department of Biology, Carleton University 1125 Colonel By Drive, K1S5B6, Ottawa, Canada; 2 School of Computer Science, Carleton University 1125 Colonel By Drive, K1S5B6, Ottawa, Canada; 3 Institute of Biochemistry, Carleton University 1125 Colonel By Drive, K1S5B6, Ottawa, Canada; 4 Institute for Research in Immunology and Cancer (IRIC), Université de Montréal, Montréal, Québec, H3C 3J7, Canada

## Abstract

Increasingly sophisticated knowledge about RNA structure and function requires an inclusive knowledge representation that facilitates the integration of independently –generated information arising from such efforts as genome sequencing projects, microarray analyses, structure determination and RNA SELEX experiments. While RNAML, an XML-based representation, has been proposed as an exchange format for a select subset of information, it lacks domain-specific semantics that are essential for answering questions that require expert knowledge. Here, we describe an RNA knowledge base (RKB) for structure-based knowledge using RDF/OWL Semantic Web technologies. RKB extends a number of ontologies and contains basic terminology for nucleic acid composition along with context/model-specific structural features such as sugar conformations, base pairings and base stackings. RKB (available at http://semanticscience.org/projects/rkb) is populated with PDB entries and MC-Annotate structural annotation. We show queries to the RKB using description logic reasoning, thus opening the door to question answering over independently-published RNA knowledge using Semantic Web technologies.

## Background

The ability to accurately capture biomolecular behaviour is critical to our understanding of cellular systems. With biophysical instruments that measure everything from bond vibrations to fluorescence as a result of molecular interactions, scientists carefully translate these observations into a set of positive statements about the entities under investigation. The set of entities, objects and relations used by scientists, through their *lingua franca*, defines a *conceptualization* of their subjects of study. The explicit commitment to a conceptualization not only enables scientists to easily share knowledge, but also permits the creation of machine-understandable knowledge bases. An ontology is an explicit specification of a conceptualization of a particular domain of knowledge [1], in which the set of objects and their relations define its scope.

In some cases, the conclusions drawn about numerous experimental results do not necessarily apply universally, but instead appear as a result of a context-dependent experimental system. Biological situational modelling [2,3] has been used as a methodology to capture this knowledge in a precise and accurate manner, so that conflicting statements about biochemical entities may be tolerated provided there exists some circumstantial qualification. Hence, a long term solution for knowledge representation in the life sciences must consider context, in addition to identity and action.

Ribonucleic acids (RNAs) are essential cellular components with significant roles in protein synthesis and gene regulation. Increasingly sophisticated knowledge about RNA structure and function is being revealed as a result of innovative biochemical investigations such as genome sequencing projects, sequence alignments, microarray analyses, structure determination and RNA SELEX experiments. Yet, our capacity to capture this knowledge by existing systems is limited in several important respects. First, RNAML [4], an XML-based exchange format for a select subset of information about RNAs, does not provide explicit formalization of the domain either from a logically or philosophical perspective. As an example, base stacking can be described with a natural language comment associated with the base-stack element, but we cannot specify a machine understandable type – what kind of thing is base stacking and what specializations of it exist (e.g. adjacent stacking or upward stacking). Second, XML Schema is primarily interested in the validation of the document structure, as opposed to the semantics of the domain terminology therein contained, thus language extensions cannot be properly validated. In contrast, RDF/OWL are formal (logic) languages which enable the explicit formalization of the domain, and as such can be used to infer new knowledge using some information system. Moreover, as languages of the Semantic Web, researchers may also publish their knowledge so as to further enhance structural and functional annotation in a machine accessible, but de-centralized manner.

Here, we describe an RNA knowledge base (RKB) for structure-oriented knowledge using RDF/OWL Semantic Web technologies. RKB extends the RNAO, an RNA ontology jointly developed with the RNA Ontology Consortium [5], and builds on other Open Biomedical Ontologies (OBO) for information content entities (e.g. PDB files, structure models), real world entities (e.g. base pairs, base stacks) and their qualities (e.g. nucleoside/sugar conformations). RKB is populated from RNA-specific PDB entries and base pairing/stacking identified by MC-Annotate. We demonstrate how the resulting knowledge base supports powerful question answering over OWL-DL ontologies using a description logic system.

## Results

This project pursued four main objectives: i) to unequivocally represent basic biochemical knowledge about nucleic acids and their structural characteristics, ii) to accurately capture the knowledge generated by a nucleic acid structural feature annotator such as MC-Annotate in such a way that it complemented other structural or functional knowledge, iii) to implement a scheme for the representation of knowledge obtained as information from a computational procedure, iv) to maximize interoperability with a set of trusted external ontologies. A high quality representation should facilitate data integration and enable question answering with a reasoning-capable knowledge base. All materials are available at the project page: http://semanticscience.org/projects/rkb/.

### RNA structure ontology

The RNA knowledge base ontology extends the RNAO and provides a core set of hierarchically organized terminology for the accurate representation of RNA and their structural features. The RKB builds on material entities and qualities as defined by the BFO upper level ontology, the RO relation ontology for reusable domain independent relations, the Information Artifact Ontology (IAO) for information content entities and ChEBI for specific chemical entities and their parts.

*Material entities* are spatially extended entities whose identity are independent and can be maintained through time. Material entity is the top level class for nucleic acids, base pairs, base stacks, chemical bonds / interactions and fiat parts of nucleic acids (nucleotide residues, sugar moieties, nucleobases) where bonds extend into another part from certain terminal atoms base pairs.

*Qualities* are categorical properties that existentially depend on, among other things, material entities. This forms the top level class for the syn- or anti- quality, a conformation (RNAO:0000123) borne specifically by the nucleoside (RKB:000027) part of a nucleotide residue (CHEBI:50319) and imparts knowledge of the orientation of its respective base and sugar parts. Similarly, the envelope conformation is a quality that is solely borne by the sugar part of a nucleotide residue.

**Figure 1:**
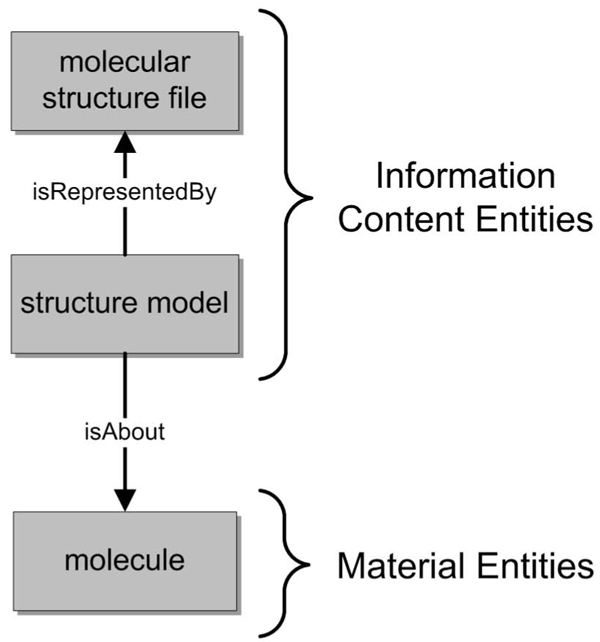
**Information content entities are about material entities in the RKB** Illustration of the RDF-based representation used to relate Information Content Entities with their corresponding Material Entities. Molecular structure files are specific manifestations of structure models therefore structure models are represented by their corresponding PDB files. Structure models are also about molecules and other real entities (atoms, base pairs, etc).

The Information Artifact Ontology’s Information Content Entities (ICEs) generically depend on at least one, but possibly more material entities. ICEs are the top level class for structure models, PDB records, coordinates and measurement values (Figure 1).

We applied the Minimum Information to Reference an External Ontology Term (MIREOT) guidelines [6] to augment the RKB ontology with relevant classes, their annotations and minimal hierarchy from the RNAO, ChEBI [7], IAO, RO and BFO. The MIREOT document consists of 45 classes from the RNAO including nucleotide base pairs, base stacks and their structural qualities and the externally connected to spatial relation, 10 classes from the IAO for molecular structure data, 24 classes from ChEBI which referred to nucleobases, nucleotides and their related sugar moieties. Conceptual overlap was captured using OWL’s class equivalence relation.

The formalization used here departs from our previous work [3] by considering a more *information-oriented* representation. Thus, instead of referring to those qualities, roles, or parts of the molecule that are involved in a situationally-dependent base pairing process, we instead consider PDB structures composed of coordinates as information content entities which are generically dependent on the material entities to which they pertain to, such as molecules and base pairs. Thus, since there is always *some* pairing/stacking process that existentially depends on the pair/stack, we name only the latter.

### RKB population

The RKB is populated with RNA structures from the PDB and results of MC-Annotate using in house scripts. The conversion of PDB structures follows our previous work on small molecule chemistry [8]. Ontology population involved three basic steps: assigning names, asserting class membership, and assigning relations between entities. Having a consistent naming scheme makes data integration from PDB entries with MC-Annotate information straightforward. Unique names were generated as valid Uniform Resource Identifiers (URI) where each name consisted of the PDB identifier followed a different naming convention for objects and qualities:

*Material Entities:*

a. Structure Model:   PDBID_cCHAIN

b. Nucleotide residue: PDBID_cCHAIN_rRESIDUE

c. Atom: PDBID_cCHAIN_rRESIDUE_aATOM

*Qualities:*

PDBID_mMODEL_cCHAIN_rRESIDUE_QUALITY

### RNA structure representation

RNA structures obtained through experimental procedures and computational model building and refinement yields a file containing information about a molecule or collection of molecules. More specifically, the file is a serialization of a data structure and contains a description of the structure model in terms of the spatial positioning of atoms as a set of coordinates in three-dimensional space. In NMR, multiple structure models may be obtained, each of which captures a significantly populated conformation. The key relation is that information content entities (e.g. coordinates and collections of coordinates) are about real world entities (e.g. atoms, molecules). Importantly, structure models provide the means by which more information about the structure and function may be determined through additional analysis.

 We used MC-Annotate over the set of PDB files that contain RNA structures to identify base pairing and base stacking in terms of their adjacency and relative orientation. A different individual was generated for each structural feature (base pairing, base stacking) of each model in the PDB file. This maintains provenance, in that entity assertions are related to the model from it was derived and also allows comparison of structures from different models.

*Base pairing*: Nucleotide base pairs may occur between any pair of nucleotide residues, and involve any number of atoms. Canonical base pairs, as described by Leontis and Westhof [9], occur as a result of the hydrogen bonding between the *edges* of nucleobases. Since edges are composed of multiple atoms and hydrogen bonds occur between pairs of atoms, Lemieux and Major [10] developed a system of finer granularity that refers to *sub-edges* or so-called *faces*. The sub-edges extend from the nucleobase along the ribose sugar and hence includes two new atoms, the O2’ and N9 or N1 for the case of purines or pyrimidines respectively. Hence, nucleotide base pairs can be represented in the RKB using either the Leontis and Westhof (LW) or the Lemieux and Major (LW+) specifications, both of which contain at least one edge or sub-edge interaction respectively. The RKB uses an “externally connected to” relation to represent the weak interactions between edges and the sub-edges in nucleotide base pairs, and this also suggests future qualitative spatial reasoning across the regions they occupy using Region Connection Calculus (RCC-8).

**Figure 2:**
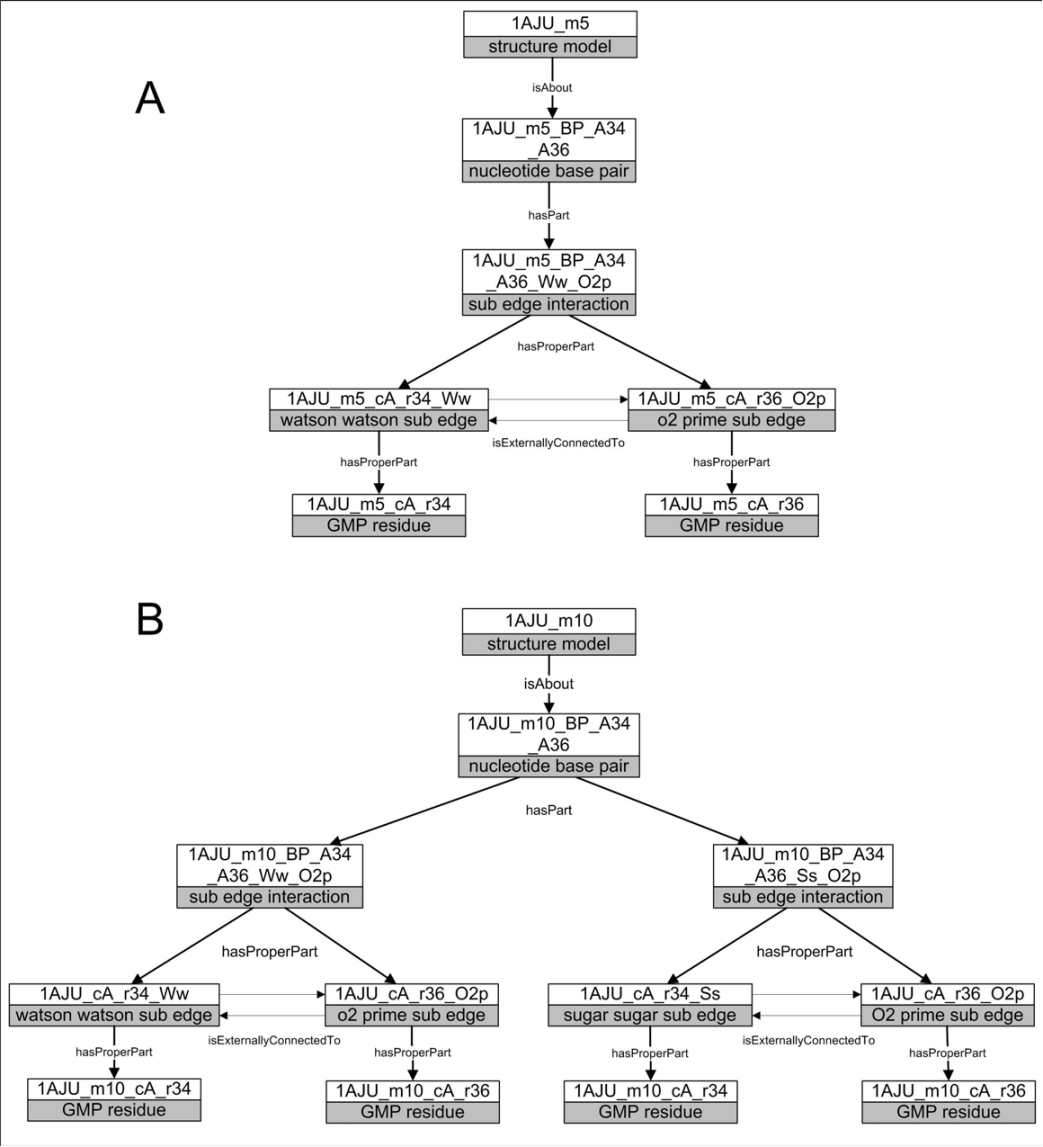
**RKB nucleotide base pairs with varying sub-edge interactions.** Illustration of the RDF-based representation of molecular structure obtained from PDB files and from structure feature analysis of MC-Annotate. (A) Structure model 5 of PDB 1AJU is about a nucleotide base pair that is composed of a sub-edge interaction between the Watson-Watson sub-edge of the guanine residue at position 34 of chain A and the O2’ sub-edge of the guanine residue at position 36 of chain A. (B) Structure model 10 of PDB 1AJU is about a nucleotide base pair between guanine residue at position 34 in chain A and guanine residue at position 36 in chain A, which is composed of two sub-edge interactions – a Watson-Watson sub-edge and O2’ sub-edge, as well as a Sugar-sugar sub-edge and the O2’ sub-edge.

Different models of the same RNA sequence may suggest flexibility through structural rearrangements. Models 5 and 10 of chain A in NMR structure PDB:1AJU suggests a difference in sub-edge interactions (Figure 2). Where model 5 shows a single sub-edge interaction between the Watson-Watson sub-edge of G34 residue and the O2’ sub-edge of the G36 residue base pair, model 10 pairs indicates two sub-edge interactions between the Ww/O2’ sub-edges and the Ss/O2’ sub-edges.

*Base stacking*: Base stacking involves a proximate spatial orientation of the top or bottom face of bases that is mitigated through weak interactions. The “externally connected to” relation stands in for non-covalent inter-molecular interactions between nucleobases. Base stacks also bear the RNAO’s “base stack base-normal orientation” and “base stack sequence adjacency” qualities for an accurate description of the relative directionality of the nucleobase normal vectors and for the description of adjacent and non-adjacent stacks.

*Puckering*: The ribose ring represents two main puckering modes, “envelope” and “twist”. The “envelope” geometry is observed when one atom is located over or below the plane formed by the four others, whereas the “twist” geometry is observed when one atom is over and another is below the plane formed by the three others. The classification of a ribose, into either geometry, is dependent on the relative position of the carbon atoms of the ribose to its C5’ atom. Hence while the carbon atoms in a ribose bear either the endo or exo qualities with respect to the plane formed by the other atoms, the ribose ring bears more specific envelop or twist qualities.

### Question answering

Example questions expressed using the Manchester OWL syntax are described below. They were formulated with the rdfs:label annotation properties using the DL Query plugin for Protégé 4 and answered using the embedded Pellet/FaCT++ reasoners. Queries (A)-(D) were performed on model 7 of PDB: 1AM0, queries (E) and (F) were performed on all models of the latter.

 (A)* How many structure models were defined in the PDB file for 1AM0:*

'structure model' that 'is represented by' some {'Molecular Structure File PDB:1am0'}

This conjunctive query with a distinguished variable returns the 8 structure models that constitute the molecular structure file 1AM0. The results of this query also illustrate how the RKB represents structure models as being representations of their corresponding PDB files.

(B) *Find all base pairs that involve a Hoogsteen edge:*

'nucleotide base pair' that 'has part' some (('hoogsteen edge' or 'part of' some 'hoogsteen edge') and externally_connected_to some ('nucleotide edge' or 'part of' some 'nucleotide edge')

This conjunctive query involves existentially qualified variables. The individual must be an instance of the *nucleotide base pair* class, but this base pair must further be specified by a *hoogsteen edge.* Yet, no individuals are asserted to be instances of Hoogsteen edges, rather since MC-Annotate generates assertions at the sub-edge level, it is also necessary to ask for any parts of the edges. The mereological inference that a sub-edge is part of an edge makes use of agglomerating sub-edge classes, where for example the “hoogsteen sub-edge” (RKB:000092) is equivalent to {“C8 sub-edge” or “hoogsteen hoogsteen sub-edge” or “hoogsteen watson sub-edge” or “bifurcated hoogsteen sub-edge”}, that establish a parthood mapping to edges, whereby the existential restrictions for the 3 edges are restricted to differing subsets of sub-edges. Finally, it is necessary to specify that the edge/sub-edge we are interested in is externally connected to another nucleotide edge/sub-edge. Two base pairs are retrieved in which the hoogsteen sub-edge is existentially known to be part of a hoogsteen edge and is also externally connected to another nucleotide edge/sub-edge.

(C)* Find all base pairs with a Hoogsteen edge that is part of a guanine residue*

'nucleotide base pair' that 'has part' some (('hoogsteen edge' or 'part of' some 'hoogsteen edge') and 'part of' some 'GMP residue [chebi:50324]' and externally_connected_to some ('nucleotide edge' or 'part of' some 'nucleotide edge'))

This query further refines Query (B), in that the Hoogsteen edge must be attached to a guanine residue and must be externally connected to another edge/sub-edge. Two base pairs are found.

(D) Find all base pairs involving a Watson-Watson sub-edge and a Hoogsteen-Hoogsteen sub-edge

'nucleotide base pair' that ('has part' some ('watson watson sub edge' and externally_connected_to some 'hoogsteen hoogsteen sub edge'))

This query aims to discover sub-edge interactions that are uniquely identified by MC-Annotate and are specified in RKB/RNAO using the *externally connected to* relation. Two results are obtained in model 7.

(E) Find all nucleotide base pairs involving at least one Hoogsteen sub edge interaction which is contained in a structure model from the PDB file 1AM0.

'nucleotide base pair' that 'has part' some (('hoogsteen edge' or 'part of' some 'hoogsteen edge') and externally_connected_to some ('nucleotide edge' or 'part of' some 'nucleotide edge')) and inv('is about') some ('structure model' that 'is represented by' some {'Molecular Structure File PDB:1am0'})

This conjunctive query with undistinguished variables (hoogsteen/nucleotide edge/part of edge, structure model) and a distinguished variable (the 1AM0 PDB file) identified 14 nucleotide base pairs out of a total of 40 across all models in the 1AM0 that involve at least one Hoogsteen edge.

(F)* Find how many structure models were defined in the pdb file for 1AM0*

'structure model' that 'is represented by' some {'Molecular Structure File PDB:1am0'}

This conjunctive query uses a distinguished variable to find all 7 structure models represented by the 1AM0 structure file.

## Discussion

### RNA on the Semantic Web

The aim of the RNA Ontology Consortium is “to create an integrated conceptual framework, an RNA Ontology (RNAO), with a common, dynamic, controlled, and structured vocabulary to describe and characterize RNA sequences, secondary structures, three dimensional structures, and dynamics pertaining to RNA function” [5]. The work described here on RNA structure and structural features provides the basis towards which other essential RNA structural and functional features may be added in the future. With contextual modelling, we represent highly dynamic features of RNA structure and function as is observed in NMR and other experiments that characterize molecular dynamics.

While the RKB requires an OWL2 compliant reasoning system to obtain all inferences, there exists substantial value in being able to publish the knowledge base as a collection of Semantic Web documents which are also accessible through a SPARQL endpoint. Future work involves provisioning the knowledge base through the Bio2RDF linked data network [11], thus enabling entity resolution and web-based interlinking between datasets.

### OWL modelling

Modelling knowledge using OWL is challenging for a number of reasons. The first is that relations between objects are binary, of the form *relation*(x,y), which precludes temporal qualification as a third argument in a ternary relation. Thus n-ary relations must be converted into n-ary objects, and this approach is exemplified in our representation of base pairing and base stacking. A second challenge is that OWL imposes certain non-structural restrictions on properties in order to remain decidable. These restrictions ensure that properties which are either transitive or part of a role chain may not be involved in cardinality restrictions (min, exactly, max), and may not also be declared as functional, inverse functional, irreflexive, antisymmetric or disjoint with another property. Role chains involving *part of* / *is about* and their inverses are useful in finding all entities that are described by information content entities. But this precludes the use of these roles in cardinality restrictions. In order to overcome this restriction, more specific sub-properties such as *has grain* / *hasquality* / *hasrole* could be used to make knowledge base assertions, and these roles are then used to make queries with cardinality restrictions. Thus cardinality restrictions can be placed in the ontology, and also in the instance base. For instance, the AA base pair class is equivalent to a Nucleotide base pair that has proper part exactly 2 adenine monophosphate residues. In this way, we can discover all such instances in RNA structure data.

### Future directions

The RKB makes use of the MIREOT scheme to incorporate selected parts of trusted external ontologies. RKB’s coverage will continue to grow alongside other resources represented with Semantic Web technologies such as the 30+ databases provided by the Bio2RDF [11] project, including UniProt [12] and the PDB. Yet a major challenge exists in ensuring that the raw linked data is massaged into more sophisticated knowledge representation schemes, such as the one described here. Ultimately, integration at both the syntactic and semantic levels across domains will allow maximum interoperability between the RKB and other relevant knowledge.

## Conclusions

The RKB facilitates RNA knowledge discovery using a set of expressive OWL ontologies instantiated with PDB structure data and annotations from MC-Annotate. The resulting knowledge base can be used for simple information retrieval and more sophisticated ontology-based knowledge discovery. Our work demonstrates the representation of information content entities such as PDB files and structure models, and how these relate to real world entities and their qualities. Continued collaboration with other members of the RNA Ontology Consortium should maximize interoperability of RNA-related information, particularly with sequence alignments, motifs and other structural and functional knowledge. Together, we will provide new avenues for biological knowledge discovery powered by the standards provided by the W3C Semantic Web effort.

## Methods

### Ontology design

The RKB ontology was designed using the OWL editor Protégé Ontology Editor (v4 Build 113) using Pellet or FaCT++ [13,14] reasoners for consistency checking.

Nucleic acid structures were obtained from the PDB [15], and MC-Annotate [10] was used to identify base pairs, base stacks, and various spatial conformations including sugar puckering. Our design approach followed a well used methodology [16]. RNA structural feature terminology was obtained from literature [17], and new terminology created to group together classes related by subsumption. Subclasses are homogenous and increasingly specialized, while each child term can be easily differentiated from its parent with clear human readable labels, accurate and concise definitions and existential / universal / cardinal axiomatic descriptions where feasible.

Upper level ontologies suggests increased interoperability and semantic coherency between domain ontologies due to grounding of the basic types of domain entities and the imposing of restrictions on the relationships that these entities may specify. Our New Upper Level Ontology (NULO), inspired by the Basic Formal Ontology (BFO) [18], offers a simple framework that enables the distinction of objects, qualities, processes and spatial regions and also features object-process, object-quality, parthood, spatial, temporal relations drawn from foundational work [19].

Classes defined in the RKB are mapped to NULO concepts. For example, when considering the horizontal plane on a ribose, and the C5’ atom is positioned to the left side, the location of the atoms with respect to the plane define either an “exo” or “endo” quality (below or above the plane, respectively) which is a quality of the corresponding atom of the ribose.

New object properties were added to further describe some of the more specific relations required in (but not restricted to) this domain. The pair *isImmediatelyAfter/isImmediatelyBefore* provides a relation between any two entities that are spatially related by adjacency. These properties permit the description of the relative positioning of nucleotide bases within a nucleic acid. They also allow for the description of the relative positioning of nucleobases that participate in either adjacent or non-adjacent stacking interactions.

## Competing interests

The authors declare that they have no competing interests.

## Authors' contributions

MD and FM conceived of the study and participated in its design. MD and JCT generated the results and drafted the manuscript. FM and MP provided technical guidance and participated in the preparation of the manuscript.
